# Vitamin D Supplementation Improves Adipose Tissue Inflammation and Reduces Hepatic Steatosis in Obese C57BL/6J Mice

**DOI:** 10.3390/nu12020342

**Published:** 2020-01-28

**Authors:** Alexandra Marziou, Clothilde Philouze, Charlène Couturier, Julien Astier, Philippe Obert, Jean-François Landrier, Catherine Riva

**Affiliations:** 1 Laboratoire de Pharm-Ecologie Cardiovasculaire (LAPEC), EA-4278, Avignon University, 84029 Avignon, France; alexandra.marziou@gmail.com (A.M.); clothilde.philouze@gmail.com (C.P.); philippe.obert@univ-avignon.fr (P.O.); 2 Aix-Marseille Université, INSERM, INRAE, C2VN, 13000 Marseille, France; charlene.couturier@univ-amu.fr (C.C.); julien.astier@univ-amu.fr (J.A.)

**Keywords:** vitamin D, inflammation, obesity, adipose tissue, steatosis, tertiary prevention

## Abstract

The beneficial effect of vitamin D (VD) supplementation on body weight gain limitation and inflammation has been highlighted in primary prevention mice models, but the long-term effect of VD supplementation in tertiary prevention has never been reported in obesity models. The curative effect of VD supplementation on obesity and associated disorders was evaluated in high-fat- and high-sucrose (HFS)-fed mice. Morphological, histological, and molecular phenotype were characterized. The increased body mass and adiposity caused by HFS diet as well as fat cell hypertrophy and glucose homeostasis were not improved by VD supplementation. However, VD supplementation led to a decrease of HFS-induced inflammation in inguinal adipose tissue, characterized by a decreased expression of chemokine mRNA levels. Moreover, a protective effect of VD on HFS-induced hepatic steatosis was highlighted by a decrease of lipid droplets and a reduction of triglyceride accumulation in the liver. This result was associated with a significant decrease of gene expression coding for key enzymes involved in hepatic de novo lipogenesis and fatty acid oxidation. Altogether, our results show that VD supplementation could be of interest to blunt the adipose tissue inflammation and hepatic steatosis and could represent an interesting nutritional strategy to fight obesity-associated comorbidities.

## 1. Introduction

Obesity is a worldwide health issue. It increases cardiovascular morbidity and impairs quality of life and consequently represents one of the leading causes of mortality. Obesity, defined by an excessive fat accumulation in adipocytes, results from an imbalance between energy intake and energy expenditure. It is caused by changes in food consumption behaviors and lifestyle [1,2]. Nowadays, according to the World Health Organization (WHO), over 1.9 billion adults are overweight and among them 650 million are obese [3]. This pathology is strongly related to metabolic disorders such as arterial hypertension, chronic low-grade inflammation, and insulin resistance that could lead to type 2 diabetes [4,5]. Obesity is also strongly linked to ectopic fat accumulation, particularly in the liver, contributing to an emergence of non-alcoholic fatty liver disease (NAFLD) [6].

Interestingly, from an epidemiologic standpoint obesity and NAFLD are commonly associated with low 25-hydroxyvitamin D (25(OH)D) plasma levels. Indeed, in prospective studies, low 25(OH)D plasma levels were associated with higher incidence of obesity in adults, children, and elderly women, it was also correlated to a higher five-year waist circumference [7]. Several cross-sectional studies have reported an inverse relationship between plasma concentrations of 25(OH)D and body mass index (BMI) [8,9,10]. However, the causality between these two parameters remains unclear [11,12]. Moreover, recent epidemiological data have also enabled the linkage of hypovitaminosis D with both obesity and NAFLD [13].

As of today, intervention studies have remained ambiguous, because some of them reported a beneficial effect on body weight management [14,15] and other studies observed no effect [16,17]. Nevertheless, preclinical studies on VD supplementation have demonstrated benefits for weight management in primary prevention in mice fed with a high-fat diet [18,19]. This was associated with the improvement of the inflammatory status in adipose tissue, characterized by a limitation of cytokines and chemokine expression by adipocytes [20], leukocyte infiltration in adipose tissue [21], and a reduction of the expression of pro-inflammatory miRNA in adipocytes and adipose tissue [22].

Nevertheless, the impact of VD supplementation on body weight reduction and associated pathologies remains poorly documented in obese mice models. A recent study reports that the body weight of obese mice was not affected by VD supplementation [23]. It is important to consider that the supplement was administered over a short period of time. The aim of this study is to evaluate the consequences of administering VD to obese mince over a longer period.

## 2. Materials and Methods

### 2.1. Animal, Diets, and Experiments

Six-week-old male C57BL/6J mice were purchased from Janvier Labs (Le Genest-Saint-Isle, France). They were kept in cages with an enriched environment maintaining controlled environmental conditions (20–23 °C; 40% humidity), and a 12-hour light/dark cycle. The mice were fed water and food ad libitum. All procedures were performed in accordance with the local research ethics committee (2017110611453051-RIVA) and the agreement of European and French Ministry of Agriculture about the care and use of laboratory animals 2010/63/EU (N°CEEA—00322.03). Mice (10-week-of age and weighing 41.55 ± 0.64 g) were randomly placed into two groups: a normal chow group (NC; *n* = 20) or a high-fat/high-sucrose diet group (HFS; *n* = 60). The NC group was fed a normal chow diet (A04, 3.1% fat, caloric value 3.339 kcal·kg^−1^, Safe, France) and water during the entire protocol of 25 weeks. The HFS group was fed a fat-enriched-dough (230HF, 60% kcal from fat with a caloric value of 5.317 kcal·kg^−1^, Safe, France) completed with drinking water containing 10% sucrose (D-Saccharose, Fisher Scientific, England) over a 10-week period to induce obesity and type 2 diabetes. After this 10-week period, the HFS group was divided in two subgroups. They were either fed with the same HFS diet or with an HFS diet supplemented with vitamin D (HFS + D; 15,000 IU·kg^−1^ cholecalciferol; customized HF230, SAFE Diet, Augy, France) over an additional 15-week period. During the experiment, body weight was measured once a week and dietary intake was assessed daily. Energy intake was calculated per cage from the amount of food and drink consumed by the animals and its caloric equivalence. At the end of the protocol, mice were fasted overnight. Animals were anesthetized, before the intracardiac puncture blood collection. The plasma was obtained by centrifuging at 3000×*g* for 15 min at 4 °C, it was then stored at −80 °C. The animals, still under anesthesia, were then sacrificed by cervical dislocation. Liver and adipose deposits (epididymal, subcutaneous, perirenal, inguinal) were entirely collected, weighed, and immediately frozen in liquid nitrogen and stored at −80 °C.

### 2.2. Insulin Tolerance Test

Seven weeks and 12 weeks after starting VD supplementation, mice were subjected to an insulin tolerance test (ITT), after they had been fasted for 6 hours. Blood was collected (5 µL) using the tail-clip method and fasting glycemia was measured using a commercially available glucometer (Accu-Check glucometer, Roche, Basel, Switzerland), in accordance with the manufacturer’s instructions. ITT were then performed after an intraperitoneal injection of insulin solution (1 U·kg^−1^), and blood glucose levels were measured from tail blood taken at the indicated times after injection: 10, 30, 60, 90, 120 minutes after the injection.

### 2.3. Biochemical Analysis

Plasma glucose and calcium (Ca**^2+^**) were analyzed using colorimetric methods (BIOLABO, Maizy, France) as non-esterified fatty acids (NEFA) (RANDOX, Crumlin, Co., Antrim, United Kingdom). Plasma insulin was measured using an enzyme-linked immunosorbent assay ELISA (ALPCO Diagnostics, New Hampshire, USA) and adiponectin was quantified using the ELISA kit (R&D Development, Minneapolis, USA) in accordance with the manufacturer’s instructions. Triglycerides (TG) were quantified both in plasma samples and in the liver using colorimetric methods (BIOLABO, Maizy, France). The Homeostasis Model Assessment of insulin resistance (HOMA-IR) index was calculated according to the following formula: fasting insulin (microU·L^−1^) × fasting glucose (mmol·L^−1^)/22.5 [24].

### 2.4. 25(OH)D Quantification in Plasma

All quantifications were performed using liquid chromatography tandem mass spectrometry (LC-MS/MS) (Hypersil Gold**^®^** C18 column, Orbitrap™ Q Exactive™ Plus system and Xcalibur™ software, Thermo Fisher Scientific, Waltham, USA) according to the protocol previously mentioned [25].

### 2.5. Histological Analysis

Paraffin-embedded tissue sections of liver and inguinal adipose tissue (iWAT) were stained with hematoxylin and eosin (H&E) using standard protocols [26]. The images were captured by a light microscope (Zeiss Axio Imager, Oberkochen, Germany) and the adipocyte area (µm^2^) was determined using (Image J) software as previously described [24].

### 2.6. RNA Isolation and qPCR

Total RNA from liver and iWAT were extracted using TRIzol reagent according to the manufacturer’s instructions (Thermo Fisher, Courtaboeuf, France). One microgram of total RNA was used to synthetize cDNA in 20 µL using random primers and Moloney murine leukemia virus reverse transcriptase (Thermo Fisher, Courtaboeuf, France). Real-time quantitative RT-PCR analyses were performed using the AriaMx System (Agilent, Santa Clara, USA) as previously described [27]. All PCR reactions were using a SYBR Green Master mix (PowerUp™ SYBR**^®^**, Thermo Fisher, Courtaboeuf, France). For each condition, expression was quantified in duplicate, and 18S rRNA was used as the endogenous control in the comparative cycle threshold (CT) method [28]. Data were expressed as a relative expression ratio. Primers sequences are presented in Supplementary Table S1.

### 2.7. Statistical Analysis

Data were expressed as the mean ± SEM. Significant differences between control and treated groups were determined using ANOVA, followed by the PLSD Fischer post hoc test using Prism6 (GraphPad Software Inc., San Diego, CA, USA). Values of *p* < 0.05 were considered statistically significant.

## 3. Results

### 3.1. Impact of High-Fat/High-Sucrose Diet and Vitamin D Supplementation on Morphological Parameters

**Figure 1:**
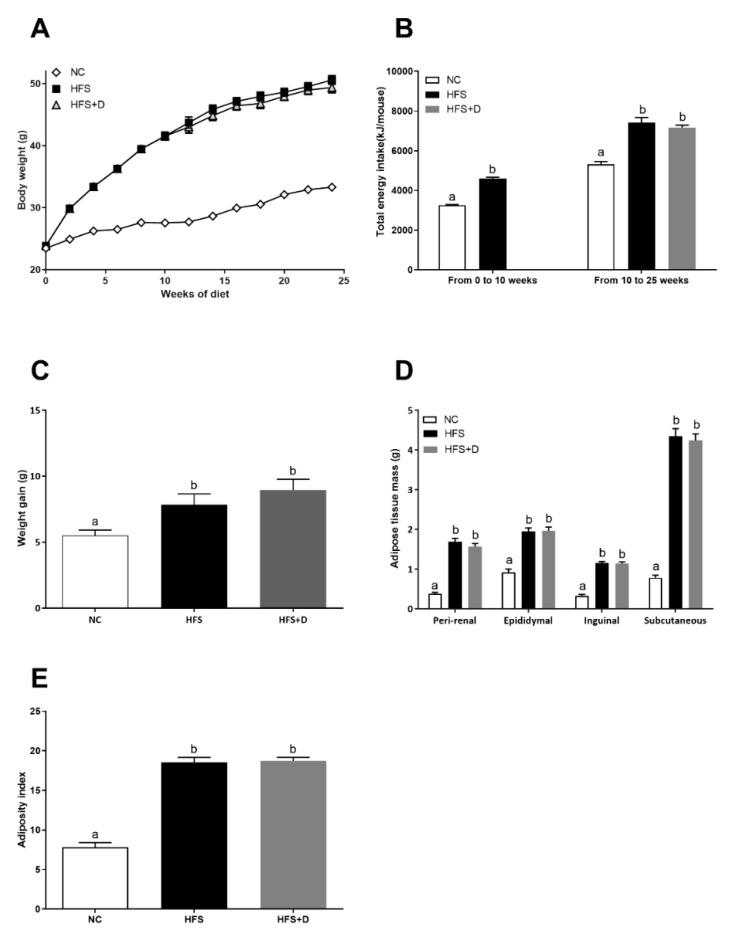
Morphological parameters of mice fed with high-fat/high-sucrose diet and vitamin D supplementation. (**A**) Body weight evolution curve of mice fed with a controlled diet (NC), high-fat/high-sucrose diet (HFS) during the first 10 weeks and then supplemented with vitamin D over a 15-week period (HFS + D). (**B**) The energy intake was quantified by measuring food intake and sucrose water every day for a period of 25 weeks following respective diet consumption. (**C**) Animal weight gain was established; it corresponds to the difference between the body weight at end of the protocol (25th week) and the body weight at the end of the 10-week HFS diet. (**D**) During the sacrifice, adipose tissues were weighed, and body composition was expressed in terms of adipose tissues’ absolute mass. (**E**) An adiposity index was calculated by calculating the ratio between the sum of perirenal, epididymal, inguinal, and subcutaneous adipose masses and body weight; values are presented as means ± SEM; values not sharing the same letter were significantly different, *p* < 0.05.

After 10 weeks of high-fat/high-sucrose (HFS) diet, body weight of HFS mice was considerably increased compared with that of the NC group (Figure 1A). In fact, energy intake was higher in the HFS group compared with that in the control group (Figure 1B). In regard to body composition, HFS consumption significantly increased adipose tissues (peri-renal, epididymal, inguinal, and subcutaneous) absolute (Figure 1D) and relative masses (Supplementary Figure S1). The adiposity index calculated by the sum of all adipose tissues relative to total body mass was increased by a factor 2 in the HFS group compared with that in the NC group. To evaluate the metabolic impact of VD supplementation, half of the mice were fed with HFS diet supplemented with VD (15,000 UI·kg^-1^ of food) (HFS + D), during an additional 15-week period. The other HFS-fed mice remained on the HFS diet. Mice that were on the HFS diet continued to gain body weight compared with those in the NC group. At the end of the 25-week protocol, the body weight as well as the adiposity index of mice supplemented with VD was not different from that of HFS-fed mice as shown in Figure 1C,F. These results were consistent with the higher energy intake in the HFS or HFS + D groups compared with that in the NC group, and there was no difference between HFS and HFS + D groups (Figure 1B).

### 3.2. Impact of High-Fat/High-Sucrose Diet and Vitamin D Supplementation on Glucose Homeostasis

**Figure 2:**
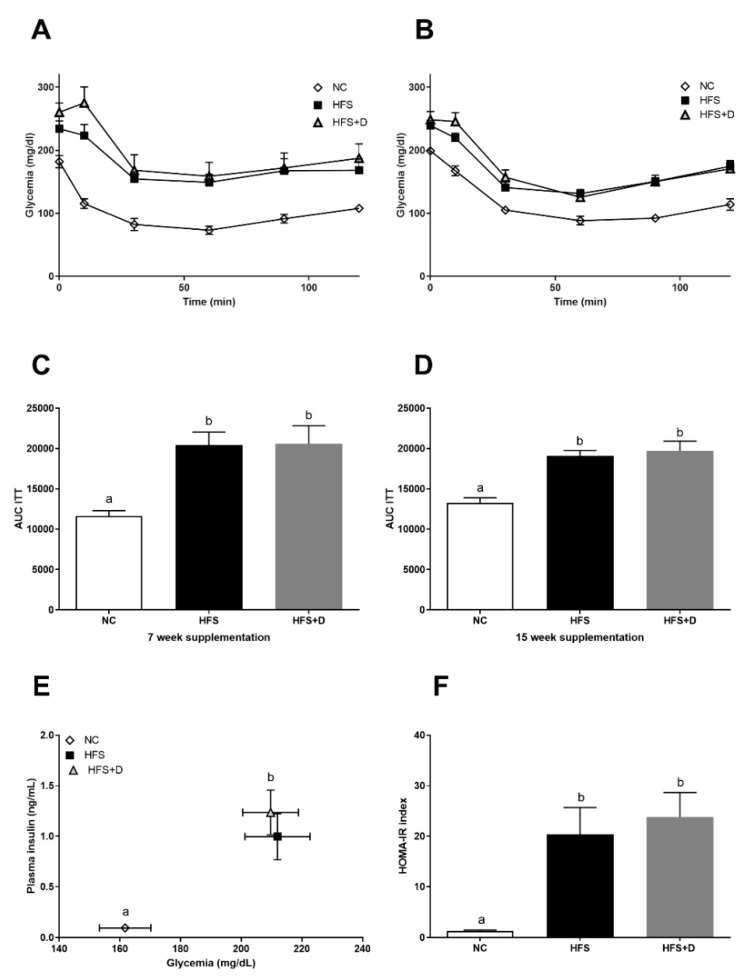
Effects of vitamin D (VD) supplementation on glucose homeostasis. (**A**) Glycemic response to insulin injection after 7 weeks of VD supplementation. (**B**) Glycemic response to insulin injection after 15 weeks of VD supplementation. (**C**) Area under the curve (AUC) calculated from the glycemic response curve after 7 weeks of VD supplementation. (**D**) AUC calculated from the glycemic response curve after 15 weeks of VD supplementation. (**E**) Quantification of plasma glucose and insulin at the end of the 25-week protocol. (**F**) HOMA-IR was calculated according to the following formula: (fasting insulin (microU·L^−1^) × fasting glucose (nmol·L^−1^)/22.5); insulin sensitivity was measured by ITT; values are presented as mean ± SEM; values not sharing the same letter were significantly different, *p* < 0.05.

Then, we evaluated the effect of VD supplementation on glucose homeostasis. At the beginning of VD supplementation, insulin resistance of mice was confirmed by ITT (Supplementary Figure S2). As expected, the HFS diet reduced insulin sensitivity (Figure 2A–D), increased glycaemia, insulinemia (Figure 2E), and HOMA-IR (Figure 2F). After 7 and 15 weeks of VD supplementation, no difference was observed in either the HFS or the HFS + D groups in terms of glycemic response. Quantification of plasma glucose and insulin at the end of the 25-week protocol (Figure 2E, Table 1) confirmed the fasting hyperglycemia and increased plasma insulin concentrations in both HFS and HFS + D groups compared with those in the NC group (Figure 2E) and HOMA-IR increased, in both HFS and HFS + D groups compared with that in the NC group (Figure 2F).

### 3.3. Impact of High-Fat/High-Sucrose Diet and Vitamin D Supplementation on Plasma Parameters

After 25 weeks, no modification of the triglyceride (TG) concentration was observed in the three groups (Table 1). The NEFA concentration was decreased in both HFS and HFS + D groups compared with that in the NC group. Adiponectin concentration had a predictable decrease in HFS-fed mice compared with that in NC-fed mice whereas unexpectedly HFS-fed mice supplemented with VD had a lower adiponectin level than both NC and HFS-fed mice. As expected, 15 weeks of VD supplementation significantly increased cholecalciferol and 25(OH)D plasma levels compared with the HFS and NC groups. Moreover, there was a slight increase of calcemia in HFS- and HFS + D-fed mice compared with that in the NC group.

### 3.4. Impact of High-Fat/High-Sucrose Diet and Vitamin D Supplementation on Inguinal White Adipose Tissue (iWAT) Biology

**Figure 3:**
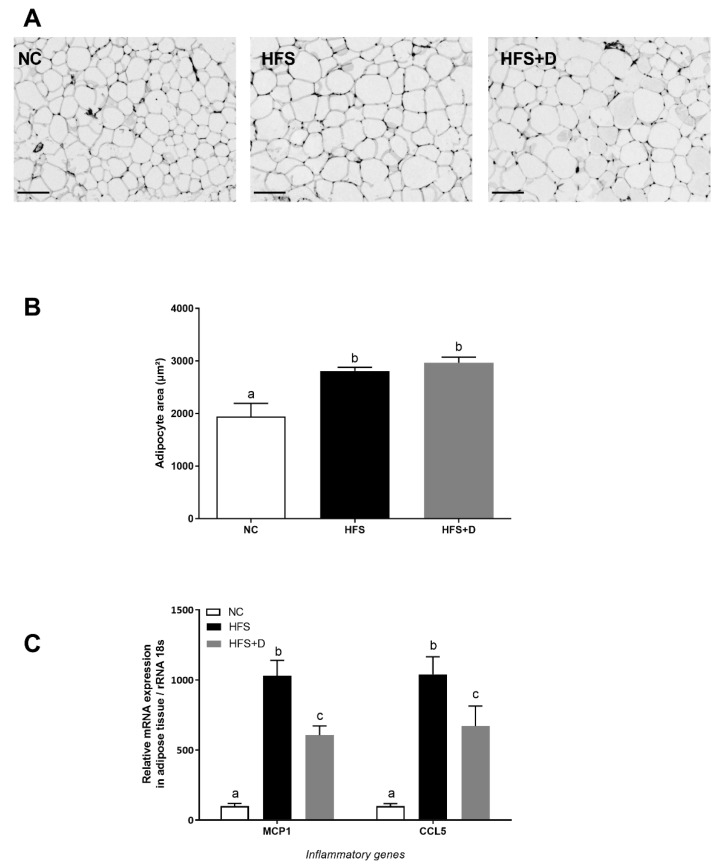
Effects of high-fat/high-sucrose diet and vitamin D supplementation on different parameters of inguinal adipose tissue. (**A**) Representative histological images of inguinal fat pads after H&E staining, taken at 40× magnification (scale bar represents 100 µm). NC: normal chow, HFS: high-fat/high-sucrose diet, HFS + D: high-fat/high-sucrose diet supplemented with vitamin D. (**B**) Adipocyte area, determined using Image J software. (**C**) Relative expression of mRNA inflammatory genes measured through qPCR and expressed relative to 18S ribosomal RNA. Values are presented as mean ± SEM. Values not sharing the same letter were significantly different, *p* < 0.05.

Histological analysis was performed to assess the effect of regimens on iWAT cellularity (Figure 3A). HFS diet induced significant enlargement of the adipocyte area compared with the NC group, while no significant change of cellularity was observed in HFS + D compared with the HFS-fed mice (Figure 3B). Furthermore, inflammatory status in iWAT evaluated by monocyte chemoattractant protein 1 (*Mcp1*) and chemokine C-C motif ligand 5 (*Ccl5*) mRNA levels revealed that their expressions were drastically increased by the HFS diet as compared with the NC group (Figure 3C), and VD supplementation significantly decreased *Mcp1* and *Ccl5* mRNA levels (41% and 36%, respectively).

### 3.5. Impact of High-Fat/High-Sucrose Diet and Vitamin D Supplementation on the Liver

**Figure 4:**
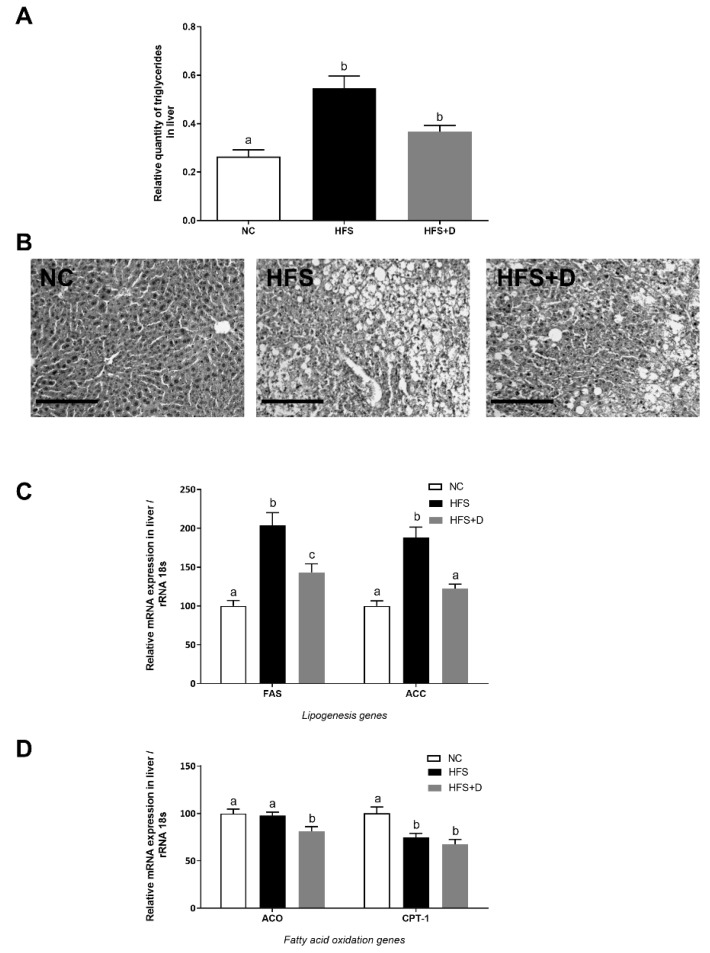
Effects of high-fat/high-sucrose diet and vitamin D supplementation on different parameters in the liver. (**A**) Quantification of triglycerides in liver tissue, related to liver mass. (**B**) Representative histological images of liver tissue after H&E staining, taken at 40× magnification (scale bar represents 200 µm). NC: normal chow, HFS: high-fat/high-sucrose diet, HFS + D: high-fat/high-sucrose diet supplemented with vitamin D. (**C**) Relative expression of mRNA genes related to lipogenesis and (**D**) fatty acid oxidation measured through qPCR and expressed relative to 18S ribosomal RNA. Values are presented as mean ± SEM. Values not sharing the same letter were significantly different, *p* < 0.05.

Triglycerides (TGs) were quantified in the liver to estimate hepatic lipid accumulation. Our results showed that the HFS diet significantly increased TGs compared with the NC diet and that VD supplementation limited TG accumulation in the liver by 33% (Figure 4A). Histological sections stained by (H&E) confirmed this result by a reduction of visible lipid droplets in VD-supplemented mice compared with that in the HFS-fed mice (Figure 4B).

The effect of VD supplementation on hepatic lipid metabolism was also studied at the molecular level. In particular, the hepatic de novo lipogenesis was evaluated by the mRNA expression of the fatty acid synthase (*Fasn*) and acetyl-coA carboxylase 1 (*Acaca*) genes (Figure 4C). Their mRNA levels were significantly increased in the HFS mice, whereas VD supplementation induced a significant decrease, by 30% and 35% for *Fasn* and *Acaca* respectively in comparison with the HFS mice. Moreover, gene expression involved in fatty acid oxidation such as the acetyl-coA oxidase (*Acox*) gene was not modified in the HFS mice whereas this level was downregulated in VD-supplemented mice (Figure 4D), while carnitine palmitoyl transferase (*Cpt1)* mRNA level was equally decreased in the HFS group and in the HFS + D group in comparison with that in NC mice.

## 4. Discussion

In the present study, we investigated the effect of VD supplementation for 15 weeks on obese C57BL/6J mice. The mice were obese as a result of an imposed high-fat/high-sucrose diet over a 10-week period prior to being given VD supplementation. We highlighted that VD supplementation did not modify the obese phenotype, adiposity, and insulin resistance in the HFS mice, but exerted an anti-inflammatory effect on iWAT and an improvement of hepatic steatosis.

The protocol implemented to generate obesity was a 10-week HFS diet, which led to a significant increase in the total body mass and adiposity index of mice and as expected was associated with hyperglycemia and insulin resistance. This diet-induced obesity and diabetes model was preferred to transgenic models for its comparison with the etiology of insulin resistance and its associated risk factors in a more physiological process. In these obese mice, 15 weeks of VD supplementation did not improve disrupted glucose homeostasis and had no impact on obesity. This observation was consistent with a recently published study that did not find evidence that body weight could be managed in obese mice via VD supplementation [23]. This is also in accordance with several clinical trials that did not report beneficial effects of VD on weight loss for overweight or obese subjects [16,29,30] and was recently meta-analyzed [31,32]. However, it is noteworthy that a study, performed on obese mice injected with 1,25(OH)2D reported a limited weight gain compared with control obese mice [33]. Such discrepancy could be due to the impact of diet-induced obesity on VD metabolism [34]; the direct administration of 1,25(OH)2D, could bypass the metabolization of cholecalciferol, and therefore be more efficient. As stated previously, it has recently been reported that obesity in humans reduced the effect of VD supplementation [35]. In the present study, we did not measure adipose tissue concentration of 1,25(OH)2D, but based on our previously reported data [34], we can speculate that this metabolite is present at a lower concentration in obese mice compared with the levels in the NC-fed mice. We can also speculate that the VD supplementation was not sufficient to reach a 1,25(OH)2D concentration able to modulate fatty acid oxidation, which is considered as a major driving force of weight loss [18,36,37].

As increased adiposity contributes to insulin resistance, insulin sensitivity was evaluated during the 25-week protocol and as expected the HFS diet induced a decreased sensitivity. Glucose metabolism parameters (glycemia, insulinemia, and HOMA-IR) also highlighted negative effects of the HFS diet, corresponding to an insulin-resistant profile [38,39]. No improvement of those parameters as well as insulin sensitivity were observed under VD supplementation. Such observation could appear to be in contradiction with other studies that demonstrate the beneficial effects of VD supplementation in patients with insulin-resistance and low VD plasma levels [40]. Indeed, the beneficial effects of VD supplementation were mainly obtained on a population with a clear disruption of glucose homeostasis on one hand [15], and on the other hand a population with VD insufficiency [40]. We have to keep in mind that our mice were not deficient in VD, as highlighted by the 25(OH)D plasma level. Thus, it is not surprising that no beneficial effects of VD supplementation were observed on glucose homeostasis parameters.

The role of adipose tissue is now not only considered as a storage site for triglycerides but also as a major endocrine organ via adipokine secretion [41]. In case of obesity, adipocyte biology is deeply modified and characterized by hypertrophy and adipokine secretion profile modification, resulting in a pro-inflammatory environment [4,42]. In agreement, our results showed that the HFS diet induced hypertrophy of adipocytes and increased mRNA levels coding for pro-inflammatory chemokines. If the VD supplementation had no effect on adipocytes morphology, it is noteworthy that VD supplementation improved the inflammatory status within the adipose tissue. Indeed, the rise of inflammatory markers (*Mcp1* and *Ccl5* mRNA levels) induced by HFS diet consumption, was reduced by VD supplementation. Such observation is clearly in line with previous demonstrations of the anti-inflammatory effect of VD, particularly at the chemokine level [20,21,43,44,45]. Even though the molecular mechanism has not been investigated in the present study, based on our previous results, we can easily hypothesize that NF-κB and/or p38 MAPK signaling are disrupted by the VD supplementation [20,21].

Interestingly, adipose MCP-1 overexpression has been reported to be associated with hepatic steatosis [46], thus special attention was given to the effect of VD on lipid accumulation and metabolism in the liver. Our results showed a protective effect of VD on HFS-induced hepatic steatosis, highlighted by a decrease of lipid droplets in the liver and a reduction of TG content. At a molecular level, VD supplementation decreased the expression of genes (*Acaca* and *Fasn*) coding for key proteins involved in hepatic de novo lipogenesis, that were induced by the HFS diet. In addition, we have also reported an induction of gene coding for proteins involved in fatty acid oxidation (*Acox*). Taken together, such modulations of genes expression could explain the limitation of TG accumulation observed in VD-supplemented mice. Similarly, a recent study depicted the role of VD supplementation on liver steatosis in a mouse model of obesity [23] and demonstrated that VD intervention significantly reduced hepatic steatosis. No effect on lipogenesis was reported in this study, and the origin of such a discrepancy is not understood at this time but could be related to the duration of the intervention.

In the human population, the role of VD in non-alcoholic fatty liver disease has recently emerged. Several observational studies reported a link between low 25(OH)D plasma levels and fatty liver diseases [13,47,48,49]. If the causality is still not clearly demonstrated in humans [50], observations on animal models, including the present study, tend to confirm such an assumption.

To conclude, even though VD supplementation did not improve body weight and insulin sensitivity, interesting data were generated in regard to the impact of VD supplementation on hepatic steatosis and adipose tissue inflammation. It is highly probable that these two occurrences are strongly linked, based on the role of adipose MCP-1 [46]. Such an assumption will require further investigations to be established. Nevertheless, VD supplementation could represent an interesting therapeutic strategy to blunt non-alcoholic fatty liver diseases.

## Figures and Tables

**Table 1:** Effect of vitamin D on mice biological parameters.

Biological Parameters	NC	HFS	HFS + D
Triglycerides (mmol·L^−1^)	1.01 ± 0.05 ^a^	1.02 ± 0.06 ^a^	1.02 ± 0.08 ^a^
Non-esterified fatty acid (mmol·L^−1^)	0.96 ± 0.05 ^a^	0.70 ± 0.04 ^b^	0.66 ± 0.04 ^b^
Adiponectin (µg·mL^−1^)	6.44 ± 0.47 ^a^	5.15 ± 0.28 ^b^	1.54 ± 0.22 ^c^
Calcium (mmol·L^−1^)	2.32 ± 0.07 ^a^	2.49 ± 0.02 ^b^	2.59 ± 0.07 ^b^
Glucose (mmol·L^−1^)	8.98 ± 0.47 ^a^	11.76 ± 0.60 ^b^	11.64 ± 0.51 ^b^
Insulin (mmol·L^−1^)	0.01 ± 0.01 ^a^	1.00 ± 0.23 ^b^	1.24 ± 0.22 ^b^
Vitamin D_3_ (ng·mL^−1^)	0.71 ± 0.20 ^a^	4.52 ± 0.81 ^a^	77.64 ± 5.86 ^b^
25(OH)D_3_ (ng·mL^−1^)	117.10 ± 3.43 ^a^	154.10 ± 5.00 ^b^	240.20 ± 12.56 ^c^

NC: normal chow, HFS: high-fat/high-sucrose diet, HFS + D: high fat/high sucrose diet supplemented with vitamin D. Values are presented as mean ± SEM. Values not sharing the same letter were significantly different, *p* < 0.05.

## Supplementary Materials

The following are available online at https://www.mdpi.com/2072-6643/12/2/342/s1, Figure S1: Adipose tissue relative mass after 25-weeks protocol; Figure S2: AUC insulin tolerance test (ITT) values of mice fed with 10 weeks of high-fat/high-sucrose diet; Table S1: Primers sequences.
