# Supplementary material for: Vitamin D Supplementation Improves Adipose Tissue Inflammation and Reduces Hepatic Steatosis in Obese C57BL/6J Mice
Source: Nutrients. 2020 Jan 28;12(2):342. doi: 10.3390/nu12020342 (PMC7071313; doi:10.3390/nu12020342)
Supplement: Supplementary file 1 [file nutrients-12-00342-s001.pdf]

Table S1. Primers sequences.

Gene		Primer sequences
m18S	Forward	CGCCGCTAGAGGTGAAATTCT
	Reverse	CATTCTTGGCAAATGCTTTCG
mMCP1	Forward	FCATCCACGTGTTGGCTCA
	Reverse	RGATCATCTTGCTGGTGAATGAGT
mCCL5	Forward	TGCAGAGGACTCTGAGACAGC
	Reverse	GAGTGGTGTCCGAGCCATA
mFAS	Forward	CATGACCTCGTGATGAACGTGT
	Reverse	TCGGGTGAGGACGTTTACAAA
mACC	Forward	GGAGTGGATGATGGTCTGA
	Reverse	GGCCTTGATCATCACTGGAT
mACO	Forward	GCTGGCCGTGTCCATAGC
	Reverse	TTATCCGTGGGTCCAAACTGA
mCPT1a	Forward	TGCCTTTACATCGTCTCCAA
	Reverse	GGCTCCAGGGTTCAGAAAGT

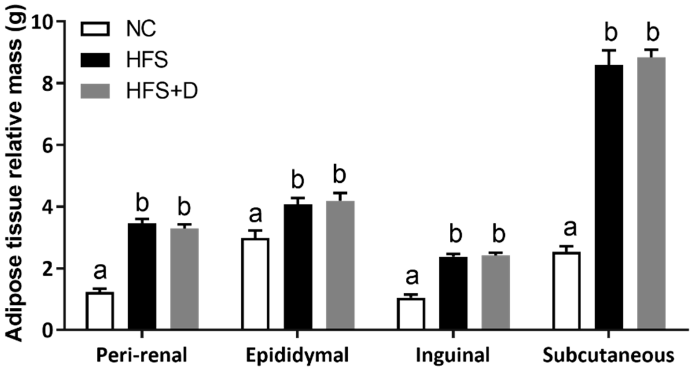

Figure S1. Adipose tissue relative mass after 25-weeks protocol. Values are presented as mean ± SEM. Values not sharing the same letter were significantly different, $p < 0.05$.

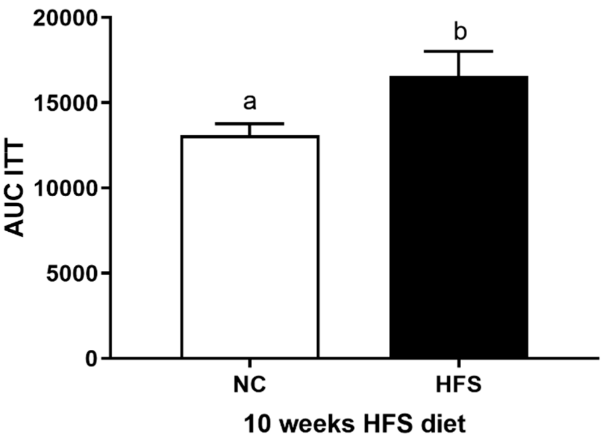

Figure S2: AUC ITT values of mice fed with 10 weeks of high fat/high sucrose diet. Values are presented as mean ± SEM. Values not sharing the same letter were significantly different, $p < 0.05$.
